# Experiences of SENSory Relearning of the UPPer Limb (SENSUPP) after Stroke and Perceived Effects: A Qualitative Study

**DOI:** 10.3390/ijerph19063636

**Published:** 2022-03-18

**Authors:** Håkan Carlsson, Ingrid Lindgren, Birgitta Rosén, Anders Björkman, Hélène Pessah-Rasmussen, Christina Brogårdh

**Affiliations:** 1 Department of Health Sciences, Lund University, 221 00 Lund, Sweden; ingrid.lindgren@med.lu.se (I.L.); christina.brogardh@med.lu.se (C.B.); 2 Department of Neurology, Rehabilitation Medicine, Memory Disorders and Geriatrics, Skåne University Hospital, 222 41 Lund, Sweden; helene.pessah@skane.se; 3 Department of Translational Medicine—Hand Surgery, Lund University, 205 02 Malmö, Sweden; birgitta.rosen@med.lu.se; 4 Department of Hand Surgery, Skåne University Hospital, 205 02 Malmö, Sweden; 5 Department of Hand Surgery, Institute of Clinical Sciences, Sahlgrenska Academy, University of Gothenburg, 405 30 Gothenburg, Sweden; anders.bjorkman@gu.se; 6 Department of Clinical Sciences, Lund University, 221 00 Lund, Sweden

**Keywords:** stroke, sensory function, sensory relearning, upper limb, qualitative study

## Abstract

Recently, it was shown that sensory relearning of the upper limb (SENSUPP) is a promising intervention to improve sensorimotor function after stroke. There is limited knowledge, however, of how participants perceive the training. Here, we explored how persons with sensory impairments in the upper limb experienced the SENSUPP protocol (combined sensory- and motor training and home exercises for 5 weeks) and its effect. Fifteen persons (mean age 59 years; 10 men; >6 months post-stroke) were individually interviewed, and data were analyzed with qualitative content analysis. An overall theme ‘Sensory relearning was meaningful and led to improved ability to perform daily hand activities’ and two categories with six subcategories emerged. The outpatient training was perceived as meaningful, although the exercises were demanding and required concentration. Support from the therapist was helpful and training in small groups appreciated. The home training was challenging due to lack of support, time, and motivation. Small improvements in sensory function were perceived, whereas increased movement control and ability in performing daily hand activities were reported. In conclusion, the SENSUPP protocol is meaningful and beneficial in improving the functioning of the UL in chronic stroke. Improving compliance to the home training, regular follow-ups, and an exercise diary are recommended.

## 1. Introduction

Sensory function of the upper limb (UL) has an important role for a person’s ability to explore and interact with the surroundings [1], as well as for movement control and the ability to use the hands in many daily activities [2]. After a stroke, which affects about 25,700 persons in Sweden [3], sensory impairments of the UL are common and approximately 40% have remaining sensory impairments in the chronic phase after stroke [4], which may negatively affect the ability to control grip force [5], to manipulate and recognize objects in the hand without support from vision [6], and the spontaneous use of the hand in everyday activities [7,8].

It is also described that persons with sensory impairments of the UL after stroke experience difficulties in many daily activities, such as personal care when showering, dressing, and tying shoelaces, in household activities when cooking, grasping objects, and using cutlery, but also during various leisure activities [9]. The activity limitations may, in turn, lead to restrictions in social roles and perceived participation [10]. Despite these problems, little attention is paid to the sensory impairments in stroke rehabilitation of the UL [9,11] and, consequently, there is a great need to develop more efficient rehabilitation interventions for the UL in persons with sensorimotor impairments after stroke [12].

One training method that may improve sensory function of the UL is sensory relearning [13,14]. The training is based on active exploration of different materials and objects with the impaired hand, with attention to sensory stimuli (i.e., touch discrimination, tactile object recognition, and proprioception). In addition, learning principles such as repetitive and intensive practice, graded progression, intermittent feedback, and calibration via vision or the unaffected hand are also used. However, as the sensory and motor system within the central and peripheral nervous system continuously interact during movements [15,16], it may be important to combine sensory and motor training (task-specific training) in rehabilitation of the UL after stroke [17].

Currently, few studies have evaluated the effect of combining sensory- and motor training of the UL after stroke [12]. Therefore, we developed a novel training protocol called ‘SENSory relearning of the UPPer limb (SENSUPP)’ after stroke where sensory relearning and task-specific training for the UL were combined [18,19]. In a recent pilot randomized controlled trial (RCT), 27 participants were randomized to either sensory relearning in combination with task-specific training (n = 15) or to task-specific training only (n = 12, control group) for 5 weeks. Briefly, we found that the sensory relearning group improved the sensory function in the hand (in terms of touch thresholds) and the performance of daily hand activities significantly more than the control group. Overall, the SENSUPP protocol was well tolerated, as assessed by predefined questions, and performed without any adverse events [20]. However, sensory relearning can be challenging and fatiguing to perform. As the training method is not routinely used in stroke rehabilitation, and research in this area is scarce, it is important to capture the participants’ own experiences of the training. A deeper knowledge could help researchers and clinicians to further develop the training protocol in order to improve functioning of the UL after stroke. Thus, the aim of this study was to explore how persons with impaired sensory function of the upper limb after stroke experienced the SENSUPP protocol and its effect.

## 2. Material and Methods

### 2.1. Research Design

This study had a qualitative research design. Semi-structured individual interviews and an inductive content analysis [21] were used to capture the participants’ experiences. By identifying differences and similarities in the material [22], this approach is appropriate to gain insight in a new research field where there is a limited number of studies [23]. The study was conducted according to the COREQ guidelines [24].

### 2.2. Participants

The 15 participants (10 men and 5 women) who had underwent sensory relearning in the pilot RCT [20] were included in this qualitative study. The participants were recruited to the pilot RCT from a rehabilitation unit at a University hospital or from outpatient healthcare settings by their treating physiotherapists or occupational therapists. Inclusion criteria for participation were: (i) sensory impairments of the UL after stroke (≤5 points in Shape-Texture Identification test, STI^TM^); (ii) ability to grasp and release a wooden block of 2.5 cm by 2.5 cm in the Box and Block test (BBT); (iii) ability to understand oral and written information; (iv) 18–80 years of age; (v) at least 6 months since stroke onset, and (vi) able to walk independently with or without an assistive device. The exclusion criterion was sensory impairment in the UL due to diseases other than stroke.

The mean age of the participants was 59 years, and the mean time since stroke was 26.5 months. Nine of them were affected in their dominant side. All had unilateral sensory and motor impairments of their UL to varying degrees, such as reduced discriminative touch, dexterity, and ability to use the hand in daily activities (Table 1).

### 2.3. A Brief Description of the SENSUPP Protocol

The SENSUPP protocol comprised sensory relearning in combination with task-specific training and was conducted as outpatient training at the stroke rehab unit at Skåne University Hospital, Sweden. The training was supervised by a physiotherapist with long experience of stroke rehabilitation, and performed in small groups of two participants, 2.5 h/session, twice a week for 5 weeks [19]. The training protocol was standardized and comprised touch discrimination of smooth and rough surfaces and materials; identification of objects of different sizes and shapes; tactile object recognition; and exercises for proprioception [19]. Exercises were calibrated via vision and/or by using the unaffected hand [6,13]. The task-specific exercises included fine and gross motor training in various activities. To enhance learning, all exercises were repetitive [25] and performed with increased difficulty [26,27]. Feedback in terms of verbal and manual guidance on the performance was provided from the therapist [28]. Home training (30 min/day) was also recommended, and the participants brought home materials for touch discrimination and/or tactile object recognition. They were also encouraged to use the affected UL as much as possible in meaningful daily activities and to reflect on the touch experiences. Overall, the training was individualized depending on the participants’ sensorimotor capacity and goals.

### 2.4. Interviews

The interviews were conducted three months after the sensory relearning was completed. The second author, a physiotherapist (IL) not involved in the training but with experience in stroke rehabilitation and qualitative research, conducted the interviews according to a semi-structured interview guide to which all authors had contributed. The guide covered questions about (i) experiences of the sensory relearning at the outpatient clinic (such as tasks, intensity, duration, and support from the therapists); (ii) the ability to perform exercises at home; and (iii) perceived effects of the training. In order to obtain as rich data as possible, follow-up questions were used such as “Can you give an example?” and “Please, describe.” The interviews were conducted from August 2017 to February 2021, and they took place in a calm and quiet room at the stroke rehab unit at Skåne University Hospital. The interviews were audio-recorded, ranged between 24 and 51 min, and resulted in 196 pages of transcribed text.

### 2.5. Data Analysis

The interviews were transcribed verbatim from the audio recordings by two of the authors (IL, HC). Thereafter, the data were analyzed as recommended by Graneheim and Lundman [21]. First, all interviews were read several times to gain familiarity with the text as a whole. Secondly, meaning units that answered the research questions were identified. Thereafter, the meaning units were condensed without losing the core meaning of the text. The condensed units were labeled with preliminary codes and sorted into subcategories. Finally, the subcategories were sorted into categories. The first (HC), second (IL), and last authors (CB) (i.e., physiotherapists with long experience of stroke rehabilitation and experience of qualitative research) interpreted the text, identified meaning units, and performed the coding and labeled the categories. The findings were validated several times until a consensus was reached. During the writing process, all authors (i.e., physiotherapists, occupational therapist, and physicians with experience of stroke rehabilitation and/or sensory relearning) discussed and refined the findings.

### 2.6. Ethics

Prior to the interviews, written informed consent was obtained from all the participants and the principles from the Helsinki Declaration were followed. The Regional Ethical Review Board in Lund Sweden (Dnr 2017/8) approved the study.

## 3. Results

**Figure 1:**
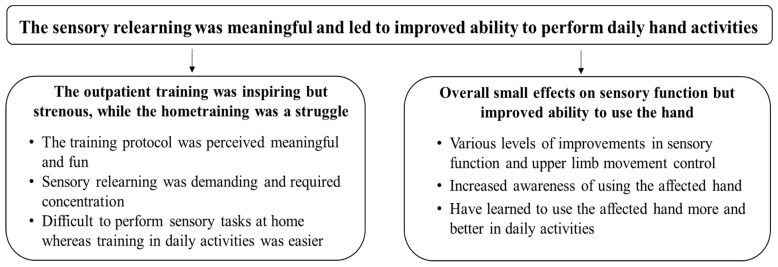
An overview of the overall theme, categories, and subcategories.

From the analysis, one overall theme ‘Sensory relearning was meaningful and led to improved ability to perform daily hand activities’ and two categories with six subcategories emerged (Figure 1). The first category ‘The outpatient training was inspiring but strenuous, while the home training was a struggle’ reflected participants’ perception of the training and included three subcategories. The second category ‘Overall small effects on sensory function but improved ability to use the hand’ reflected their perceived effects of the training and included three subcategories. To validate the findings, the results are illuminated by quotations from the participants.

### 3.1. The Outpatient Training Was Inspiring but Strenous, While the Home training Was a Struggle

Overall, the participants experienced the sensory relearning as very positive with a sufficient intensity and duration. Many expressed that the exercises were relevant and varied with a progression in difficulty. The exercises, however, required concentration, which, for some, led to a transient fatigue. Feelings of happiness and satisfaction were perceived when the tasks could be performed successfully, but also frustration and disappointment when objects could not be identified. Many enjoyed training with others and found the support from the therapist helpful. They said the home training was more challenging due to lack of support, time, and motivation.

#### 3.1.1. The Training Protocol Was Perceived Meaningful and Fun

The participants thought that the training was positive and included meaningful and relevant exercises tailored to their needs. Coming to the outpatient clinic for training was inspiring. In addition, being able to use the affected hand in a better way or in activities that had been impossible before gave motivation to continue the training.

*…I thought it was fun, it was fun to come here these days and then [do] the same at home. You feel like you have to keep it up… It was stimulating for the entire psyche and everything, it was…*

(P15)

Two training sessions per week were perceived as sufficient. Some became tired after 2.5 h due to the relatively high training intensity. The duration of 5 weeks was adequate, even if someone expressed a desire for a longer training period. One person had a wish that the training should have come earlier after the stroke onset. Overall, the exercises were perceived as well-structured and varied. The participants emphasized the importance of individualized training clearly linked to their capabilities. The level of difficulty of the exercises and the gradual increase in difficulty was experienced well adapted to their ability, even though one person with mild sensory impairments asked for more challenging training.

*Some exercises were more difficult than others and the level of difficulty gradually increased. As I managed the exercise a bit better, they increased it, so it was always at a level… challenging enough to try to feel what kind of object it was.*

(P4)

Support and feedback from the physiotherapist were helpful. It was important to obtain clear instructions as well as individual guidance and feedback on the performance. The participants also said that it was helpful to be continuously reminded to reflect on the object’s surfaces and properties during the training.

*…along the way we were given plenty of reminders, I feel. Think about this, do you feel anything, is it heavy or light, is it hot or cold, how does the surface feel on this, we were constantly given reminders to like, raise our awareness…*

(P5)

Another aspect that contributed to the appreciation of the training was that the participants trained together with another person in a similar situation. Therefore, they had the opportunity to exchange experiences, thoughts, and reflections, and to support each other.

#### 3.1.2. Sensory Relearning Was Demanding and Required Concentration

Even if the participants experienced that the sensory training was demanding and required a high degree of concentration, they understood that it was an important part of the training protocol. It was strenuous for the brain to identify the properties of the objects, leading to physical and mental fatigue. Regularly taking short breaks during the training was important. However, even if some participants had to rest at home after the training, no one experienced the exercises as too strenuous.

*It was strenuous for the brain, trying to categorize what kind of object it was… is it a soft or smooth surface… it was really difficult to push the brain like that, but it was really good.*

(P4)

The ability to manage the sensory tasks were influenced by the participant’s hand motor function. They said that it could be difficult to control the hand movements, manipulate objects, and discriminate between different materials without vision. Larger objects were, however, easier to identify than small ones where you are more dependent on active finger movements.

*…I got to have different objects in my hand without seeing what it was and had to try to identify what it was. It’s really difficult when you can’t manipulate the objects around in your hand and feel it.*

(P15)

Some participants described a frustration when they could not discriminate between different textures or objects without the help from vision. They became irritated when they could not feel the object in the affected hand but could immediately do so with their unaffected hand.

#### 3.1.3. Difficulty in Performing Sensory Tasks at Home, Whereas Training in Daily Activities Was Easier

Performing the home training with the received material for sensory training was perceived as challenging by many participants. They were less motivated to perform touch discrimination and tactile object recognition exercises at home as it was difficult to relate them to meaningful everyday activities. In addition, some had quickly learned to identify the materials they had brought home and they said that it was hard to move on with the training themselves without support from a therapist. Others described a lack of time or difficulties in disposing their time, but also difficulties in prioritizing the home training.

*…I felt like I had no motivation to do it (train at home). I couldn’t really relate it to being meaningful or not, I just felt like I’d rather do something else.*

(P14)

Many tried to use the affected hand in daily activities at home, for example, when tying shoelaces, buttoning, eating with cutlery, unscrewing bottle caps, and carrying objects. One participant also expressed that he was trying to perform some activities without vision. To facilitate the home training, a document with a clear description of the exercises was suggested.

*It should’ve been written down on a piece of paper, so you could see what kind of stuff (exercises) it was… (then) you would’ve put more effort into it to enjoy the training at home.*

(P9)

### 3.2. Small Effects Overall on Sensory Function but Improved Ability to Use the Hand

The perceived effects of sensory relearning varied among the participants. Few experienced an improved sensory function of their affected UL, whereas several participants perceived an improved movement control. In addition, many described an increased awareness of the hand and that they had learned to use the hand more in everyday activities.

#### 3.2.1. Various Levels of Improvements in Sensory Function and Upper Limb Movement Control

While most participants experienced that the sensory function was at the same level as before the training, a few perceived a gradual sensory improvement over time, particularly in identifying larger objects such as a glass or a bottle.

*But [the training] has given me.., I could more easily feel things when I grab them. I can feel if I’m grabbing a glass or a loaf of bread, I can connect it in my brain somehow.*

(P12)

Improvements in controlling and adjusting the UL movements in various activities were also described. Some perceived an improved ability to adjust grip force, which allowed them to rely on their arm more and it made it easier to handle cutlery or carry a glass of water. Others mentioned that it was easier to control the arm with improved timing in gross movements, for example, when opening and closing a door or when playing boules.

*Now, when I’m at a restaurant cutting and such, I don’t need to think that much about how I’m cutting. But at first [before the sensory training], I just ripped it [the piece of meat] apart. …So, something about the training has made me feel like I have better control of how hard to grasp things…*

(P1)

#### 3.2.2. Increased Awareness of Using the Affected Hand

The sensory relearning had also led to an increased awareness of the affected UL. Participants described that they were more aware of their capacity, and how they could use the affected hand in daily life in ways they had not been able to before the training.

*It feels like the brain is more aware—that now you have to use the left hand too… For example when you grab a plate or turn on the faucet [and you think]: Can you use the left one? …and not only use the right one which is automatically preferred.*

(P2)

However, using the affected hand in everyday activities required concentration and many had learned that it was important to use vision to compensate for the impaired sensory function. Even if someone perceived that the affected hand was used more automatically, a majority mentioned that they had to remind themselves to use the hand in daily activities. They still had to think actively what the hand should do, but they expressed hope that it would become more automatic in the future.

*[I think about] making sure that I work a lot more with my right [hand]. I try to open doors with my right [hand], comb my hair with my right [hand], shave with my right [hand], so that I don’t just rely on my left [hand]… it could be better, but I’ve done it a lot more consciously since the training than I did before. But it’s not automatic.*

(P3)

#### 3.2.3. Learning to Use the Affected Hand More and Better in Daily Activities

After the sensory relearning, the participants had learned to use their affected hand more in meaningful everyday activities. They perceived improvements in tasks that required both gross motor and fine motor skills. An improved ability was experienced in personal care, for example, when washing, combing, putting on and taking off socks, and tying shoelaces. Cooking and eating food was also easier, for example, using cutlery, cutting food, and drinking from glasses. Other activities that were easier to perform were emptying the dishwasher, turning lights on and off, and opening and closing a door. Some also expressed an improved ability to use the affected hand in bimanual activities such as vacuuming, making the bed, and hanging laundry.

*…I get things out from the dishwasher, have started combing my hair [with the affected hand], I’m vacuuming with both my hands… I’ve started eating with cutlery in both hands… this thought hadn’t even struck me before, even trying these things.*

(P11)

## 4. Discussion

This qualitative study explored how persons with sensorimotor impairments of the UL after stroke experienced the SENSUPP protocol (consisting of combined sensory- and motor training and home exercises for 5 weeks). Our main findings are that the outpatient training was perceived as inspiring and meaningful, although the exercises were demanding and required concentration. Support from the therapist was helpful and training in small groups appreciated. The home training was perceived as challenging by the participants due to lack of support, time, and motivation. Overall, small improvements in sensory function were perceived, whereas improved movement control and ability in performing daily hand activities were reported.

The participants appreciated the outpatient training very much. They thought that the exercises were inspiring, relevant, meaningful, and adapted to their hand function and capacity. The importance of including meaningful activities in stroke rehabilitation is well known [29,30], which increase participants’ motivation and adherence to the training [31]. However, even though the sensory relearning was inspiring, the training was strenuous as the tasks were challenging and required a high degree of concentration. Being able to identify textures and objects without vision placed great demands on the sensorimotor function of the hand. Even though the SENSUPP protocol was demanding, and the participants became tired during the exercises, no one experienced fatigue as a major problem during the training. Similar findings were also described by Merlo et al. [32] and Turville et al. [14], where participants managed to perform sensory relearning of the UL despite experiencing fatigue. This suggests that people after stroke could manage intensive rehabilitation, but therapists need to adjust the training so that it is tailored to the individual’s needs and capacity.

Overall, the SENSUPP protocol with 25 h of training in total was perceived sufficient regarding frequency and duration. In studies where sensory and motor training were combined, the total amount of training ranged between 7 and 72 h with better functional recovery of the UL the longer the training duration [12]. The total amount of training in our protocol was based on previous studies after stroke [33,34] and indicates that two training sessions/week for 5 weeks was adequate even if some participants preferred to have a longer training period. Currently, it is unknown exactly how the optimal training protocol should be set-up for the best possible effect, and thus more studies are needed.

The sensory relearning in the present study was based on current neurobiological learning principles, including repetitive and intensive practice with gradually increased difficulty and with extrinsic feedback [13]. All these aspects are known to be important for learning and essential elements for improved outcomes in rehabilitation [35]. Support from the therapist when performing the tasks was also perceived as important among our participants. This is in line with previous studies [14,36], showing that encouragement from the therapist can facilitate participants’ motivation and satisfaction with the rehabilitation [37,38]. Training in small groups was also perceived as very positive, and having contact with a person in a similar situation and receiving supportive learning from others who are in a similar situation was important. Benefits with group training have been highlighted in other studies [36,39], and sharing experiences with others could also have an essential role for a person’s empowerment [40]. These aspects are important to consider when sensory relearning for the UL after stroke is provided.

While the outpatient training was perceived as inspiring and meaningful, the participants struggled with their home training. In particular, it was hard to perform the sensory tasks as it was difficult to relate them to meaningful activities. In addition, the challenges to make the training more difficult at home affected their motivation and adherence to the training. Instead, they focused on trying to use the affected hand more in everyday activities, such as personal and household activities. Similar findings have been described by Byl et al. where participants expressed that they had difficulties in disciplining themselves to practice at home [41]. Using an exercise diary might have increased their motivation to train at home and to monitor the progress [42]. Home training is an important component in stroke rehabilitation [42,43], as it is a possible way to increase the time spent in active practice and to connect the training to real-life environments. Thus, this component in the SENSUPP protocol needs to be further improved in the future.

Regarding the effects of the SENSUPP protocol, several participants perceived benefits of the sensory relearning, although the effects on sensory function were small. Most of the participants expressed that the sensory function was mainly unchanged. These experiences are in line with the objective outcomes in our pilot RCT [20], showing only a significant difference in one (touch thresholds) of three sensory modalities after the training. Possible explanations for the small changes could be that many of the participants had a severe sensory impairment of the UL and were in the chronic phase post-stroke. Applying the SENSUPP protocol for persons with a mild to moderate sensory impairment and in the early post-stroke phase may have been more effective in improving sensory function, but this needs to be further investigated in future studies.

Even though the effect on sensory function was modest, several of our participants experienced an improved movement control, both to control arm movements and to control grip force after the sensory relearning. They also described that they had learned to use the hand better in various activities, such as carrying a glass of water, eating with cutlery, and playing boules. This implies that they were more confident in using their hand in everyday activities, and that they may have overcome the so-called ‘learned non-use phenomenon’ [44]. It has previously been shown that sensory impairments could lead to difficulties in maintaining attention to the affected hand, leading to a learned non-use phenomenon [17]. The findings are also in line with our recent pilot RCT [20] where an improved use and satisfaction of performance of using the hand in daily activities were found after 5 weeks of sensory relearning and at 3 months follow-up. The repetitive and intensive practice in the SENSUPP protocol with gradually increasing difficulty and feedback may have contributed to the improved ability to use the hand in everyday activities. Other studies have also reported an increased use of the affected UL in daily activities both after sensory training [14] and after sensorimotor training [45], indicating that improving the functioning of the UL after stroke is important with a high attention to the affected hand and applying learning principles in the rehabilitation.

### 4.1. Methodological Considerations

The interviews in the present study and the inductive content analysis [23] enabled us to gain an increased understanding on participants’ experience of sensory relearning and perceived effects, which is helpful in knowing whether the SENSUPP protocol needs to be further developed. A strength with the study is that all 15 participants from the sensory relearning group [20] were interviewed, and that age, gender, time since stroke onset, and degree of sensorimotor impairments in their affected UL varied among the participants, which is an advantage in qualitative studies [22]. A semi-structured interview guide with follow-up questions was used during the interviews, and an independent person who had not been involved in the training conducted all the interviews, which we believed made it easier for the participants to talk freely about their experiences. To enhance the credibility of the results, subcategories and categories were discussed by the authors during the analysis process to reach a consensus about the interpretation and confirm that they illustrate the data. To further illuminate the findings, representative quotations from the participants were used. Another strength is that the authors had different professions and pre-understanding of the patient group and sensory relearning, which enriched the interpretation of the results. However, the study also had some limitations. The participants were interviewed at the time of their three-month follow-up, and therefore, some of the participants may have had some difficulties in recalling their experiences of the training protocol. In addition, as the participants in our study took part in sensory relearning in the chronic phase after stroke, our findings cannot be generalized to persons in the early phase post-stroke.

### 4.2. Clinical Implications

The clinical implication of this study is that the SENSUPP protocol with intensive combined sensory- and motor training twice a week during five weeks was well tolerated and perceived as meaningful by the participants and could be used as outpatient training for persons with UL sensorimotor impairments after stroke. Individualized tasks based on participant’s capacity, gradual increase in difficulty, and guidance and feedback from the therapist is important to consider in order to increase participants’ motivation to the training. Training in small groups is appreciated and could further improve compliance to the training as participants can share experiences and support each other. To improve adherence to the home training, the exercises should be meaningful and structured, and an exercise diary is recommended.

## 5. Conclusions

Sensory relearning is experienced as a strenuous but inspiring and meaningful training method. Individualized outpatient training combined with guidance and feedback from a therapist and training in groups are appreciated. Home training could be challenging due to lack of support, time, and motivation. Even though sensory relearning led to small effects on sensory function, an increased movement control and ability to use the hand in daily life was described. Our findings imply that the SENSUPP protocol is meaningful and beneficial in improving the functioning of the UL in chronic stroke. However, following-up the participants’ training at home and using an exercise diary are recommended.

## Figures and Tables

**Table 1:** Characteristics of the 15 participants.

Variables	Values
Age	
Years; mean (SD)	59 (11.9)
Gender	
Male (n)	10
Female (n)	5
Time since stroke	
Months; mean (SD)	26.5 (27.5)
Type of stroke	
Ischemic (n)	11
Hemorrhage (n)	4
Side of paresis	
Right	9
Left	6
Dominant hand affected	
Yes	9
No	6
Discriminative touch	
STI; median (min-max)	0 (0–4)
Gross manual dexterity	
BBT; median (min-max)	28 (1–48)
Ability to use the hand in daily activities	
MAL AOU; median (min-max)	2.1 (0.8–4.5)
MAL QOM; median (min-max)	1.7 (0.6–4.3)

Abbreviations: SD = standard deviation, n = number, SWM = Semmes–Weinstein monofilament, STI = Shape-Texture Identification test (0–6 points), BBT = Box and Block test, (no. of blocks/min)), MAL AOU = Motor Activity Log Amount Of Use (0–5 points), MAL QOM = Motor Activity Log Quality Of Movement (0–5 points) [18].

## Data Availability

All data were archived according to the Swedish Act concerning the Ethical Review of research involving humans to attain confidentiality and are available upon reasonable request.
